# Stories of a Country Doctor

**Published:** 1890-09

**Authors:** 


STORIES OF A COUNTRY DOCTOR. By WILLIS P. KING, M. D., Member of the American Medical Association; Member and Ex-President of the Missouri State Medical Association; Assistant-Chief Surgeon of the Missouri Pacific Railway Company, etc., etc. With illustrations by T. A. Fitzgerald. Small octavo. Pp. 397. Kansas City: Hudson-Kimberly Publishing Company. 1890.

This book is written by an eminent physician, who is also an accomplished surgeon, and is full of such experiences as only a country doctor can boast. The anecdotes provoke laughter in spite of their sometimes pathetic turn, for they relate to a life that is incident to the development of our western civilization. In this progressive march towards the occident, physicians have played an important part. They have not only ministered to the sufferings of the people, and smoothed the pillows of the dying, but they have often performed the functions of civil office, and have otherwise contributed to the education and improvement of the communities where their lots have been cast.

Dr. King is a graceful writer, with a fund of information that he has told in a pleasant way, and his book will afford agreeable companionship at many a fireside, where it will be read by the families that are fortunate enough to possess it.

